# Antibiotics Differentially Modulate Lipoteichoic Acid-Mediated Host Immune Response

**DOI:** 10.3390/antibiotics9090573

**Published:** 2020-09-03

**Authors:** Marquerita Algorri, Annie Wong-Beringer

**Affiliations:** School of Pharmacy, University of Southern California, Los Angeles, CA 90089, USA; algorri@usc.edu

**Keywords:** *Staphylococcus aureus* bacteremia, sepsis immunoparalysis, antibiotics, immunomodulation, lipoteichoic acid

## Abstract

In *Staphylococcus aureus* bacteremia, our group has shown that a dysregulated balance of pro- and anti-inflammatory cytokine response biased towards an immunoparalysis phenotype is predictive of persistence and mortality, despite receipt of antibiotics. Certain antibiotics, as well as lipoteichoic acid (LTA) released from *S. aureus*, can modulate immune response ex vivo. Here, we evaluated the effects of three anti-staphylococcal antibiotics (vancomycin, tedizolid, and daptomycin) on the expression of cytokines and cell surface markers of immune activation (TNFα, HLA-DR) and immunoparalysis (IL-10, PD-L1) in human peripheral blood mononuclear cells (PBMC) exposed to high (10 μg) and low (1 μg) doses of LTA. Results suggested a dose-dependent relationship between LTA and induction of anti- and pro-inflammatory immune responses. Differential antibiotic effects were prominently observed at high but not low LTA condition. Vancomycin significantly induced IL-10 and TNFα expression, whereas daptomycin had no effects on cytokine response or expression of cell surface receptors. Tedizolid increased TNFα and modestly increased HLA-DR expression, suggesting a stimulatory effect. These findings suggest that anti-staphylococcal agents differentially alter LTA-mediated immune cell activation status and cytokine response, providing support for future clinical studies to better elucidate the complexities of host–microbial–antibiotic interaction that can help direct precision therapy for *S. aureus* bacteremia.

## 1. Introduction

The majority of sepsis-related deaths occur more than five days after onset of sepsis [1]. Late-stage mortality has been attributed to a dysregulated host immune response predominated by increased production of anti-inflammatory cytokines that confers an immunoparalysis state leading to persistent primary infection or development of secondary infections in a subset of patients [2,3]. Notably, downregulation of human leukocyte antigen DR-isotype (HLA-DR) is the most prominent and frequently measured marker of immunoparalysis in sepsis [4]. HLA-DR is a major histocompatibility (MHC) class II cell surface receptor present on monocytes and dendritic cells that is responsible for presenting antigens to CD4+ T cells, thereby initiating and activating the adaptive immune response. An additional marker of interest that has been shown previously to be strongly associated with increased risk of mortality in patients with sepsis is programmed cell death ligand 1 (PD-L1) on monocytes, which communicates with the inhibitory program cell death receptor (PD-1) and initiates T cell apoptosis [5].

*Staphylococcus aureus* bacteremia (SAB) is the most common bacterial cause of sepsis in intensive care unit (ICU) patients and is associated with significant morbidity and mortality [6,7]. Despite receipt of antibiotic therapy, persistent growth of the bacterium in blood for at least three days after start of antibiotic therapy occurs in 37% of patients and is associated with 16% increased risk of death for each day of persistent bacteremia [8]. Our group has reported that an early onset of dysregulated host immune response, as evidenced by high IL-10/TNFα ratio, was predictive of persistence and 30-day mortality in SAB [3,9]. High IL-10/TNFα ratio suggests a shift towards immunoparalysis, leading to bacterial persistence.

Lipoteichoic acid (LTA) is an essential component of the cell wall of *S. aureus* that is released spontaneously during normal growth, as well as following antibiotic exposure, with notable increases in release observed in the presence of cell-wall active antibiotics [10,11]. Of interest, LTA has been shown to affect host immune response through its interactions with immune cells by exerting immunostimulatory effects through the induction of pro-inflammatory cytokine release, whereas others have found LTA to be a potent inducer of IL-10 production resulting in reduction of HLA-DR expression and inhibition of T cell activation via PD-1/PD-L1 [12,13,14,15,16,17].

In clinical practice, the current approach to managing SAB relies on the selection of antibiotics based on organism identification and susceptibility alone, without specific regard to the host immune response. Published studies have demonstrated that some antibiotics can exert immunomodulatory effects on host cells, separate from their direct inhibitory and killing action upon susceptible pathogens. In this study, we hypothesize that host immune response is affected by LTA release from *S. aureus* and that the LTA-mediated immune response is differentially impacted upon exposure to anti-staphylococcal antibiotics from different pharmacologic classes: vancomycin (VAN), tedizolid (TED), and daptomycin (DAP). Our ultimate goal is to aid clinicians in the selection of an antimicrobial regimen that maximizes treatment success for *S. aureus* bacteremia by harnessing both its antimicrobial and immunomodulatory actions, specific to the individual patient’s immune response to the infecting pathogen.

## 2. Results

### 2.1. Purified LTA from S. aureus Differentially Affected Expression of Cytokine and Cell Surface Markers in a Dose-Dependent Manner in Human PBMCs

**Figure 1:**
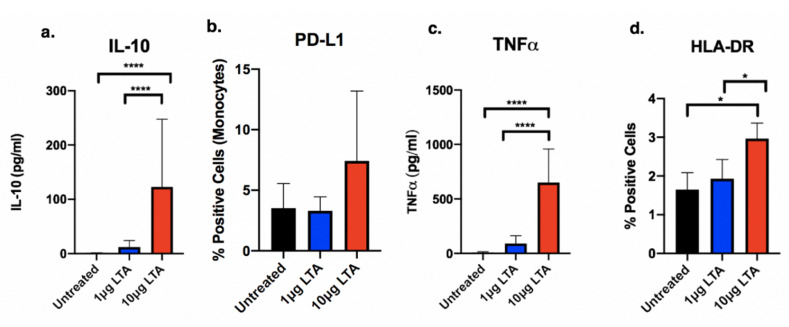
Effects of high (10 μg/mL) and low (1 μg/mL) exposure levels of purified *S. aureus* LTA on (**a**) IL-10, (**b**) PD-L1, (**c**) TNFα, and (**d**) HLA-DR expression in human PBMCs (*n* = 4). Protein expression was determined using either flow cytometry (PD-L1, HLA-DR) or ELISA (IL-10, TNFα. Data are displayed as means with error bars depicting standard deviation. Brackets indicate statistical comparisons between treatments. * *p* < 0.05, **** *p* < 0.0001.

We used two different concentrations of LTA (high: 10 μg/mL; low: 1 μg/mL) to stimulate human PBMCs obtained from healthy volunteer donors. Previously published in vitro data demonstrate that LTA release with and without antibiotics typically ranges between 1 and 10μg, with variations among clinical *S. aureus* strains [10,11]. We examined four relevant sepsis markers to assess host immune response phenotype: IL-10, TNFα, HLA-DR, and PD-L1 (Figure 1). Increased expression of PD-L1 and IL-10 would suggest an immunoparalysis phenotype characterized by decreased pathogen recognition and dampened pro-inflammatory response. Conversely, enhanced expression of TNFα and HLA-DR would be expected in activated, pro-inflammatory cells as these functions are associated with pathogen detection, activation, and microbial clearance. The results demonstrate that at baseline, IL-10 and TNFα are produced in minimal quantities, and there is low expression of PD-L1 on the cell surface in the absence of stimuli. As PBMCs are a mixed cell population containing T cells, B cells, monocytes, dendritic cells, and NK cells, there is a relatively smaller proportion of cells that expresses HLA-DR (1.6% of PBMCs HLA-DR+ at baseline).

In comparison to baseline, exposure to high concentrations of LTA significantly increased expression of IL-10 and TNFα, with a minimal observed increase in PD-L1 expression. HLA-DR expression also significantly increased with high LTA versus low LTA (*p* = 0.01) and untreated cells (*p* = 0.05) (2.96% vs. 1.93% vs. 1.65% HLA-DR+ PBMCs, respectively). When compared to low LTA, there was a clear concentration-dependent effect in PBMCs, with high LTA causing significantly greater response with respect to TNFα and HLA-DR expression. While high LTA appeared to have a greater stimulatory effect, it was accompanied by a compensatory increase in IL-10 and PD-L1 (IL10: 123 pg/mL vs. 12.23 pg/mL and PD-L1+: 7.41% vs 3.31% at high and low LTA, respectively). Conversely, low LTA minimally affected either TNFα or IL-10 production compared to untreated controls.

### 2.2. Antibiotics Exert Drug- and Dose-Dependent LTA-Mediated Effects on Cytokine and Cell Surface Marker Expression in Human PBMCs

**Figure 2:**
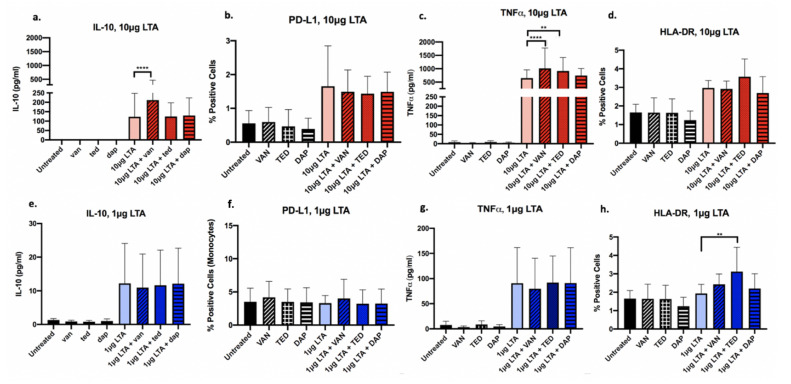
Expression of select immune markers in human PBMCs (*n* = 4) exposed to antibiotics and 10 μg/mL (high) (**a**–**d**) or 1 μg/mL (low) (**e**–**h**) purified *S. aureus* LTA. Antibiotics include VAN, vancomycin; TED, tedizolid; DAP, daptomycin. Protein expression was determined using either flow cytometry (PD-L1, HLA-DR, TLR2) or ELISA (IL-10, TNFα). Antibiotics alone and untreated cells were used as controls. Data are displayed as means with error bars depicting standard deviation. Brackets indicate statistical comparisons between treatments: ** *p* < 0.01; **** *p* < 0.0001.

Following co-stimulation with LTA and antibiotics (VAN, TED, or DAP), PBMC expression of IL-10, TNFα, HLA-DR, and PD-L1 was analyzed using ELISA or flow cytometry. Figure 2 depicts the drug- and LTA-dose-dependent effects observed in PBMCs treated with VAN, TED, or DAP. In the presence of high-concentration LTA, VAN exposure significantly increased IL-10, and TNFα cytokine production had no effect on HLA-DR and modestly downregulated PD-L1 surface expression of PBMCs. TED significantly increased TNFα cytokine release. DAP had no significant effects on any of the markers measured. Thus, it appears that in the setting of high-dose LTA, TED appears to have the greatest immunoactivating potential, through its actions on TNFα release. While VAN increased TNFα, it also demonstrated anti-inflammatory properties through its increase in IL-10 release, while DAP appeared to have no effect on the selected anti- or pro-inflammatory markers, contributing a neutral effect on the host immune response.

Conversely, at the low LTA exposure level, antibiotics had a less pronounced effect in comparison to high LTA on all markers of interest, indicating that with greater LTA-mediated immune stimulation, antibiotics have an enhanced capacity to exert immunomodulatory effects. When stimulated with low LTA, VAN demonstrated a modest increase in HLA-DR and PD-L1 cell surface expression. TED significantly increased HLA-DR (*p* = 0.003). Regardless of high or low LTA exposure, DAP had no significant effects on protein production for any of the markers of interest examined. Overall, TED appeared to exert the least immune dampening effect upon exposure of PBMCs to low LTA relative to VAN and DAP due to its ability to increase HLA-DR and TNFα. Minimal to no effect on antibiotic-mediated cytokine response was observed under low LTA condition.

## 3. Discussion

In this study, we compared antibiotics from unique pharmacologic classes on their potential to shift the balance of pro- and anti-inflammatory host immune responses in the presence of two different concentrations of LTA. Two concentrations of LTA were used to mimic the differential LTA-releasing abilities of clinical strains, as well as to simulate different bacterial burden during infections. Our results demonstrated that antibiotics have greater potential to modulate host immunity in the presence of high versus low concentration LTA. Vancomycin trended towards exerting an anti-inflammatory effect with increased IL-10 production, whereas tedizolid displayed pro-inflammatory effects, as evidenced through its stimulatory actions on TNFα and HLA-DR. Daptomycin largely had no effect on host immune response in the presence of LTA. 

Prior studies have demonstrated differential immunomodulatory potential of antibiotic agents on cytokine response in monocytes from healthy donors when stimulated with LPS or bacteria ex vivo. A multitude of experimental models have been employed varied by stimuli, concentrations, or inoculum tested, incubation time of monocytes with stimuli, antibiotic agent and concentrations tested, and specific cytokines measured [18,19,20]. A concentration-dependent immunomodulatory effect is observed for some agents on pro-inflammatory cytokines, while other agents exerted no effect [18,19,20,21,22]. Specifically, linezolid, an oxazolidinone that is pharmacologically similar to tedizolid, has been shown to induce pro-inflammatory cytokine mRNA production in THP-1 monocytes [23]. However, the effect is not well characterized, as other studies examining linezolid’s effects on immune cells in the presence of microbial components have shown differential results that may be due to donor-specific variables or interactions with microbial toxins [24]. While LTA remains a key component of gram-positive organisms, including *S. aureus*, it is important to note that many other microbial components contribute to the host immune response during in vivo infection. Thus, the results shown here using purified LTA are a limitation of this study.

Overall results from our study indicate that immune response from PBMCs was dependent on LTA concentration and drug-specific exposure. Our results suggest that TED may be preferred for use in patients who exhibit an immunoparalysis phenotype, as TED-mediated enhancement in TNFα cytokine release and HLA-DR expression was observed, without impacting IL-10 expression. It is possible that in the context of in vivo infection, TED exerts its immunomodulatory effects indirectly through suppression of *S. aureus* toxin production [25]. In contrast, VAN significantly induces IL-10 production which may tip the balance towards an immunoparalysis state. DAP largely has no effect on the markers of interest at either LTA exposure level, which may render this antibiotic as a neutral treatment option; selection for treatment would be based primarily on its direct antimicrobial activity. In the setting of low LTA exposure, antibiotics had less robust effects on cytokine or cell surface marker expression in PBMCs suggesting that therapy selection would be based primarily on the direct antimicrobial activity of the antibiotic agent.

Importantly, given that anti-staphylococcal antibiotics from different drug classes modulate host cytokine response in a distinct LTA-dose-dependent manner, future studies evaluating clinical efficacy of different treatment regimens should take into account the agent’s antibacterial effect and immunomodulatory potential along with the patient’s immunophenotype based on measurements of immune markers (e.g., pro- and anti-inflammatory cytokines), as well as LTA release profile of the infecting strain. For individualized patient therapies, tempering of TNFα response, while preserving HLA-DR expression may be beneficial for patients infected with low LTA-releasing strains, whereas patients infected with high LTA-releasing strains may benefit from avoidance of antibiotics that exert an immune dampening effect. Additional studies are necessary to further develop a model for precision antibiotic therapies, which are currently underway in our lab to better elucidate the complexities of host-microbial-antibiotic interaction that drive outcomes of *S. aureus* bacteremia. Findings from our study support the rationale for moving towards a precision medicine approach in infectious disease therapy by illustrating complex antibiotic-specific immunomodulatory differences that likely impact patient outcomes and survival.

## 4. Materials and Methods

### 4.1. Isolation of Peripheral Blood Mononuclear Cells (PBMCs)

PBMCs were obtained from whole blood of healthy volunteer donors. All donors gave informed consent to participate in the study, as approved by the Institutional Review Board (IRB) for the University of Southern California. All research conducted in this study was conducted in compliance with IRB-approved protocol and international human-subjects research regulations. 

PBMCs were isolated using SepMate™ PBMC Isolation tubes and Lymphoprep™ density gradient (STEMCELL Technologies, Vancouver, BC, Canada). Briefly, whole blood was diluted 1:1 in phosphate buffered saline (PBS) and 2% fetal bovine serum (FBS) and layered over the density gradients in the separation tubes and centrifuged for 10 min at 1200× *g*. Isolated cells were washed in PBS three times and resuspended in fresh Roswell Park Memorial Institute (RPMI) 1640 medium with 10% FBS. 

Following isolation, PBMCs were seeded at a density of 2.5 × 10^5^ cells per well in RPMI 1640 with 10% FBS. Cells were stimulated as described below.

### 4.2. Cell Culture and Stimulation

*LTA Stimulation conditions*. Cells were either untreated, stimulated with antibiotics alone, stimulated with 1 μg or 10 μg of commercially obtained purified LTA from *S. aureus* (Invivogen, San Diego, CA, USA), or stimulated with 1 or 10 μg of LTA plus antibiotics. The following anti-staphylococcal agents were selected for testing to represent different pharmacologic classes: vancomycin (VAN), tedizolid (TED), and daptomycin (DAP). Concentrations of antibiotic used were the minimum inhibitory concentrations (MICs) for vancomycin, tedizolid, and daptomycin against the common and well-characterized LAC USA300 community-acquired MRSA clinical strain (1.5 μg/mL VAN, 2 μg/mL TED, and 0.25 μg/mL DAP, respectively). MICs were determined using broth microdilution testing.

*Timepoint measurement.* The timepoints at which flow cytometry, cytokine measurements, and flow cytometry experiments were conducted at 4 h (data not shown) and 24 h, with more prominent effects demonstrated at 24 h. Cytokine measurements were collected at 24 h to compensate for both peak release and degradation and to allow for direct comparison with flow cytometry data.

### 4.3. Cytokine Quantification by ELISA

Cell culture supernatants were collected in triplicate from PBMCs following 24 h of stimulation with LTA. Cell-free supernatants of the samples were recovered by centrifugation at 400× *g* for 10 min to remove any residual cells or debris and stored at −80 °C until assayed for cytokine concentrations. Release of IL-10 and TNFα was determined using MesoScale Discovery multiplex ELISA and analyzed in duplicate (MesoScale Discovery, Gaithersburg, MD, USA). The accompanying SECTOR Imager SI2400 and MSD Workbench software were used for analysis. The lower limit of detection for IL-10 and TNFα was 0.04 pg/mL, as per manufacturer instructions. Samples with values below the limit of detection were assigned a value of 0 pg/mL.

### 4.4. Immune Cell Surface Marker Expression by Flow Cytometry

PBMCs were analyzed for HLA-DR and PD-L1 expression using flow cytometry following 24 h of stimulation. Cells were stained and examined for expression of HLA-DR by staining with a BD Quantibrite combined antibody reagent, consisting of an anti-human HLA-DR monoclonal antibody, clone L243, labelled with PE and an anti-human CD14 monoclonal antibody, clone MφP9, labeled with PerCP-Cy™5.5 (BD Biosciences, San Jose, CA, USA). PD-L1 expression was assessed by staining with an APC-labeled anti-human PD-L1 monoclonal antibody clone MIH1 (BD Biosciences, San Jose, CA, USA). Monocytes were selected from the PD-L1 stained cells by size distribution and granularity (FSC and SSC gating). Data were collected using the BD LSR Fortessa X-20 and analyzed using FlowJo software (FlowJo, LLC, Ashland, OR, USA). Total number of cells analyzed per tube included 100,000 donor PBMCs.

### 4.5. Statistical Analysis

Statistical analysis was performed using Graphpad Prism version 8.0 (Graphpad Software, San Diego, CA, USA). Data are represented through mean and standard error. One-way ANOVA or two-way ANOVA with post-hoc corrections, where applicable, were utilized to assess statistical differences between treatment groups. *p* values ≤ 0.05 were considered significant.

## References

[1] Hotchkiss R.S., Monneret G., Payen D. (2013). Sepsis-induced immunosuppression: From cellular dysfunctions to immunotherapy. Nat. Rev. Immunol..

[2] Minejima E., Bensman J., She R.C., Mack W.J., Tran M.T., Ny P., Lou M., Yamaki J., Nieberg P., Ho J. (2016). A Dysregulated Balance of Proinflammatory and Anti-Inflammatory Host Cytokine Response Early During Therapy Predicts Persistence and Mortality in Staphylococcus aureus Bacteremia. Crit. Care Med..

[3] Hotchkiss R.S., Monneret G., Payen D. (2013). Immunosuppression in sepsis: A novel understanding of the disorder and a new therapeutic approach. Lancet Infect. Dis..

[4] Monneret G., Venet F. (2014). Monocyte HLA-DR in sepsis: Shall we stop following the flow?. Crit. Care.

[5] Shao R., Fang Y., Yu H., Zhao L., Jiang Z., Li C. (2016). Monocyte programmed death ligand-1 expression after 3–4 days of sepsis is associated with risk stratification and mortality in septic patients: A prospective cohort study. Crit. Care.

[6] Tong S.Y., Davis J.S., Eichenberger E., Holland T.L., Fowler V.G. (2015). Staphylococcus aureus Infections: Epidemiology, Pathophysiology, Clinical Manifestations, and Management. Clin. Microbiol. Rev..

[7] Vincent J.-L., Rello J., Marshall J., Silva E., Anzueto A., Martin C., Moreno R., Lipman J., Gomersall C.D., Sakr Y. (2009). International Study of the Prevalence and Outcomes of Infection in Intensive Care Units. JAMA.

[8] Minejima E., Mai N., Bui N., Mert M., She R.C., Nieberg P., Spellberg B., Wong-Beringer A., Mack W.J. (2020). Defining the Breakpoint Duration of Staphylococcus aureus Bacteremia Predictive of Poor Outcomes. Clin. Infect. Dis..

[9] Salas D.E., Minejima E., Wu J., Fang C., Wang J., She R., Nieberg P., Wong-Beringer A. (2017). Staphylococcus aureus Bacteremia in Patients not Meeting Sepsis Criteria: Clinical Features, Host Immune Response and Outcomes. J. Clin. Med. Ther..

[10] Lotz S., Starke A., Ziemann C., Morath S., Hartung T., Solbach W., Laskay T. (2006). Beta-lactam antibiotic-induced release of lipoteichoic acid from Staphylococcus aureus leads to activation of neutrophil granulocytes. Ann. Clin. Microbiol. Antimicrob..

[11] Van Langevelde P., Van Dissel J.T., Ravensbergen E., Appelmelk B.J., Schrijver I.A., Groeneveld P.H.P. (1998). Antibiotic-Induced Release of Lipoteichoic Acid and Peptidoglycan from Staphylococcus aureus: Quantitative Measurements and Biological Reactivities. Antimicrob. Agents Chemother..

[12] Fournier B. (2013). The function of TLR2 during staphylococcal diseases. Front. Microbiol..

[13] Rockel C., Hartung T. (2012). Systematic Review of Membrane Components of Gram-Positive Bacteria Responsible as Pyrogens for Inducing Human Monocyte/Macrophage Cytokine Release. Front. Pharmacol..

[14] Lotz S., Aga E., Wilde I., Van Zandbergen G., Hartung T., Solbach W., Laskay T. (2004). Highly purified lipoteichoic acid activates neutrophil granulocytes and delays their spontaneous apoptosis via CD14 and TLR2. J. Leukoc. Biol..

[15] Hattar K., Grandel U., Moeller A., Fink L., Iglhaut J., Hartung T., Morath S., Seeger W., Grimminger F., Sibelius U. (2006). Lipoteichoic acid (LTA) from Staphylococcus aureus stimulates human neutrophil cytokine release by a CD14-dependent, Toll-like-receptor-independent mechanism: Autocrine role of tumor necrosis factor-α in mediating LTA-induced interleukin-8 generation. Crit. Care Med..

[16] von Aulock S., Morath S., Hareng L., Knapp S., van Kessel K.P., van Strijp J.A., Hartung T. (2003). Lipoteichoic acid from Staphylococcus aureus is a potent stimulus for neutrophil recruitment. Immunobiology.

[17] Wang J., Roderiquez G., Norcross M.A. (2012). Control of Adaptive Immune Responses by Staphylococcus aureus through IL-10, PD-L1 and TLR2. Sci. Rep..

[18] Morikawa K., Watabe H., Araake M., Morikawa S. (1996). Modulatory effect of antibiotics on cytokine production by human monocytes in vitro. Antimicrob. Agents Chemother..

[19] Franks Z., Campbell R.A., de Abreu A.V., Holloway J.T., Marvin J.E., Kraemer B.F., Zimmerman G.A., Weyrich A.S., Rondina M.T. (2013). Methicillin-resistant Staphylococcus aureus-induced thrombo-inflammatory response is reduced with timely antibiotic administration. Thromb. Haemost..

[20] Pichereau S., Moran J.J.M., Hayney M.S., Shukla S.K., Sakoulas G., Rose W.E. (2012). Concentration-dependent effects of antimicrobials on Staphylococcus aureus toxin-mediated cytokine production from peripheral blood mononuclear cells. J. Antimicrob. Chemother..

[21] Plevin R.E., Knoll M., McKay M., Arbabi S., Cuschieri J. (2016). The Role of Lipopolysaccharide Structure in Monocyte Activation and Cytokine Secretion. Shock (Augusta Ga.).

[22] Thallinger C., Rothenburger M., Marsik C., Wuenscher S., Popovic M., Endler G., Wagner O., Joukhadar C. (2008). Daptomycin Does Not Exert Immunomodulatory Effects in an Experimental Endotoxin Model of Human Whole Blood. Pharmacology.

[23] Bode C., Muenster S., Diedrich B., Jahnert S., Weisheit C., Steinhagen F., Boehm O., Hoeft A., Meyer R., Baumgarten G. (2015). Linezolid, vancomycin and daptomycin modulate cytokine production, Toll-like receptors and phagocytosis in a human in vitro model of sepsis. J. Antibiot..

[24] Wang J., Xia L., Wang R., Cai Y. (2019). Linezolid and Its Immunomodulatory Effect: In Vitro and In Vivo Evidence. Front. Pharmacol..

[25] Yamaki J., Synold T., Wong-Beringer A. (2011). Antivirulence Potential of TR-700 and Clindamycin on Clinical Isolates of Staphylococcus aureus Producing Phenol-Soluble Modulins. Antimicrob. Agents Chemother..

